# Increased low frequency fluctuation in the brain after acupuncture treatment in CSVDCI patients: A randomized control trial study

**DOI:** 10.3389/fnins.2023.1125418

**Published:** 2023-02-27

**Authors:** Nan Yang, Sina Chen, Shuxue Liu, Shuiqiao Ling, Lidian Chen

**Affiliations:** ^1^Fujian University of Traditional Chinese Medicine, Fuzhou, Fujian, China; ^2^Zhongshan Hospital of Traditional Chinese Medicine, Zhongshan, Guangdong, China

**Keywords:** cognitive function, acupuncture, small vessel disease, fMRI, brain activity

## Abstract

**Background:**

Cerebral small vessel disease (CSVD) is one of two cognition-impairing diseases. Acupuncture (Acu) is a flexible treatment with few adverse effects and is thus widely used to treat neurological problems.

**Methods:**

We recruited a total of 60 patients and assigned them to two groups (*n* = 30 each group). During the study, some participants were excluded by quality control, and a total of 44 subjects (25 Acu and 19 controls) were completed to investigate the therapeutic efficacy of acupuncture on CSVD cognitive impairment (CSVDCI). The following demographic and clinical variables were compared between the two groups: gender, age, education, smoking, alcohol, Montreal cognitive assessment (MoCA), symbol digit modalities test (SDMT), verbal fluency test (VFT), digit span task (DST), Boston naming test (BNT) scores, and amplitude of low-frequency fluctuation (ALFF) under the typical band (0.01–0.08 Hz). Mixed effect analysis was utilized to test for differences between the two groups before and after the treatment.

**Results:**

Following acupuncture treatment, the Acu group scored higher on MoCA, SDMT, VFT, DST, and BNT compared to controls (*P* < 0.05). The brain regions showing substantially greater ALFF values in the Acu group were the right inferior temporal gyrus, left middle occipital gyrus, left superior occipital gyrus, left insula, bilateral postcentral gyrus, right superior parietal gyrus, right cerebellum, right precuneus, and right precentral gyrus (*P* < 0.005, no correction). The ALFF values in the right inferior temporal gyrus (*P* = 0.027), left middle occipital gyrus (*P* = 0.005), left superior occipital gyrus (*P* = 0.011), and right superior parietal gyrus (*P* = 0.043) were positively associated with MoCA.

**Conclusion:**

We found that acupuncture modulates the functional activity of temporal, occipital, and parietal regions of the brain in CSVDCI patients.

## 1. Introduction

CSVDCI is the most common cause of vascular cognitive impairment (VCI), accounting for approximately 74% of all occurrences (Rao et al., 2009). It is not commonly known that CSVDCI is an important subtype of VCI due to its quiet onset and lack of prominent clinical features (Brookes et al., 2014), also representing the primary clinical manifestation of CSVD. CSVDCI shows a similar pattern of cognitive decompensation to the VCI, which is characterized by reduced executive function, attention, and information processing speed, with relatively intact memory function in the early stages and gradual development of dementia (Chen et al., 2019). In addition, cognitive impairment worsens with the development of the disease, which severely impacts patient quality of life.

Early management of CSVDCI can enhance cognitive performance and patient quality of life, while partially slowing the course of cognitive decline. Several conservative treatments, including the use of an acetylcholinesterase inhibitor and N-methyl-d-aspartate (NMDA) receptor antagonist were proposed for the symptomatic treatment of dementia (Arvanitakis et al., 2019). However, a limited number of targeted drugs effectively improved cognitive function in CSVDCI patients (Pantoni, 2010), with minimal efficacy for dementia (Bath and Wardlaw, 2015). Interestingly, acupuncture has been widely used in China as a complementary alternative treatment for dementia, and also been accepted for VCI treatment in Western medicine (Ji et al., 2021). The main advantage of acupuncture is the lower incidence of adverse effects that characterize pharmaceutical approaches (NIH Consensus Conference, 1998; Kim et al., 2019). Importantly, clinical randomized trials demonstrated the short-term impact of acupuncture on cognitive function in VCI patients (Yang et al., 2014; Yang et al., 2019; Huang et al., 2021).

With an increasing understanding of the etiological basis of CSVD in the elderly population, inflammatory responses have been associated with its development and progression (Li et al., 2020). Wang et al. demonstrated that acupuncture attenuates inflammation-related cognitive impairment in experimental vascular dementia (VD) by inhibiting the miR-93-mediated TLR4/MyD88/NF-κB signaling pathway (Wang et al., 2020). In addition, acupuncture reduces oxidative stress and inflammation associated with TXNIP, plays a neuroprotective role in VD rats (Du et al., 2018), enhances cognitive function and induces neuroprotective effects against inflammation in CCH rats by activating α7nAChR and the JAK2-STAT3 pathway (Cao et al., 2021).

Various ancient and modern acupuncture publications showed the Shenting (GV24) and Baihui (GV20) are vital distal acupoints associated with the cure of dementia, dizziness, headache, among other brain diseases. Huang et al. (2015) conducted a meta-analysis study that included 1,637 subjects with post-stroke cognitive impairment (PSCI), and found that integrating Shenting and Baihui acupuncture with computer-assisted cognitive training significantly improves attention deficits in stroke patients. Similar results were found in a randomized controlled trial of 2 × 2 factorial design conducted by Yang S. et al. (2014).

Resting-state functional magnetic resonance imaging (MRI) has been extensively employed to investigate the functional mechanisms underlying a variety of neurological diseases, and may also provide insights on the ability of acupuncture to improve cognitive performance (Cai et al., 2018). Measurements such as functional connectivity (FC) and degree centrality (DC) were created to mimic the brain network (Park and Friston, 2013). Zang et al. (2007) suggested ALFF to estimate regional brain activity and found it could represent the activity of different brain regions at the resting state. In addition, abnormal ALFF levels were found in people with cognitive problems and abnormal brain function, a powerful determinant of cognitive decline (Li et al., 2021; Wang et al., 2021; Zhang J. et al., 2021), and different brain regions, including the parietal, insular and cingulate regions. This is significantly correlated with cognitive function in patients with subcortical vascular cognitive dysfunction, which may lead to decreased cortical activation (Li et al., 2015). CSVDCI patients with cerebral microbleeds (CMBs) have altered spontaneous brain activity of the default, sensorimotor, and fronto-parietal lobe networks, that may impact potential neurophysiological mechanisms of intrinsic brain activity (Feng et al., 2021).

Since 1990, an increasing number of studies used imaging to explore the physio-pathological mechanisms of acupuncture for the treatment of disease (Dhond et al., 2007). Acupuncture improves cognitive function in patients with Parkinson and increases ALFF values of the default network, visual network, and insular lobe. This has led to the hypothesis that acupuncture can activate the cerebellum-thalamus-cortex loop by regulating the spontaneous activity of the brain in key regions, a neurophysiological mechanism to improve cognitive dysfunction (Li Z. et al., 2018). Moxibustion therapy can improve the cognitive function of patients with mild cognitive impairment by adjusting the ALFF values of the default, visual and subcortical networks (Lai et al., 2022), and might thus reveal the brain regions involved in cognitive function improvement through acupuncture.

Here, we examined the differences in ALFF values between the Shenting/Baihui acupoints and conventional drug treatment in CSVDCI patients, before and after treatment (in 12 weeks), to uncover the associated neural mechanisms.

## 2. Materials and methods

### 2.1. Participants

CSVDCI patients were enrolled at the Zhongshan Hospital of Traditional Chinese Medicine from July 1st 2017 to July 30th 2019. The protocol was approved by the research ethics committee of the Zhongshan Hospital of Traditional Chinese Medicine (reference: 2017ZSZY-LLK-219). We recruited CSVDCI patients at the neurology outpatient and inpatient departments. All participants signed an informed consent form prior to enrollment.

Patients with the following conditions were considered eligible: (i) age between 40 and 80 years, (ii) comply with diagnostic imaging criteria for cerebral small vessel disease and vascular cognitive impairment, (iii) MoCA score between 10 and 26, (iv) not receiving regular acupuncture treatment for the recent six months.

Patients with the following conditions were excluded: (i) cognitive dysfunction caused by macrovascular, cardiogenic cerebral embolism, (ii) patients with severe speech, vision, or hearing impairments or mental disorders that impact cognitive examinations, (iii) cognitive dysfunction caused by neuropsychological disorders (e.g., depression), (iv) illiterates that could not cooperate with cognitive examinations, (v) prior alcohol and drug abuse experience, (vi) combination of serious diseases, including of the cardiovascular, hepatic, nephrology, endocrine system and hematopoietic systems, (vii) participating in other clinical trials.

The CSVD patients were diagnosed according to the Neuroimaging Standards for Research into Small Vessel Disease (Wardlaw et al., 2013). Specifically, the diagnostic standard for imaging of CSVD included: (i) Recent small subcortical infarct: Axial views showing an infarct diameter smaller than 20 mm, which could be larger than 20 mm in the coronal or sagittal views, (ii) Lacunes of presumed vascular origin: round or ovoid, 3–15 mm in diameter, distributed in subcortical regions, filled with the same signals as cerebrospinal fluid (CSF), (iii) white matter hyperintensity (WMH) of presumed vascular origin: abnormal brain white matter (WM) signals, lesions of variable size, showing a high signal on the T2-weighted or T2-weighted FLAIR images. (iv) Perivascular space: the signal of perivascular space was the same as that of the CSF in all MRI sequences. The shape was linear when the image plane ran parallel to the blood vessels and round or oval when running perpendicular to the vessels, usually smaller than 3 mm in diameter, (v) Cerebral microbleeds, which were defined as the following changes in the images obtained with T2*-weighted gradient-echo sensitive to magnetizing effects. For example: (1) small round or oval, clear boundary, homogeneity, lack of signal focus; (2) diameter of 2–5 mm (maximum 10 mm) and lesion surrounded by the brain parenchyma; (3) brain atrophy: reduced brain volume not associated with specific focal lesions, such as trauma and cerebral infarction.

The sample size was estimated using the Gpower3.1 software. The MoCA total score was used as the main impact indicator. Based on previous studies (Wang et al., 2016), which estimated the MoCA difference for VCI patients treated with acupuncture as 5.5 ± 2.2, and the MoCA difference for the control group as 3.1 ± 1.8. The Gpower3.1 software estimated the effect value for acupuncture to improve cognitive function in VCI patients to be 1.194045, whereby we set the α value to 0.01, the Power (1-β) value to 0.9, and the effect value to 1.194045, which was calculated using a sample size of 23 cases per group. With a shedding rate of 20%, we predicted a total sample size of 56, with 28 cases per group.

A total of sixty patients were enrolled after screening for eligibility, and were randomly allocated to either the acupuncture or conventional treatment groups. At the baseline, all patients underwent fMRI. We removed 11 and 5 patients from the conventional and acupuncture groups, respectively, due to excessive head motion or rejection of the second fMRI scan.

### 2.2. Protocol

**FIGURE 1:**
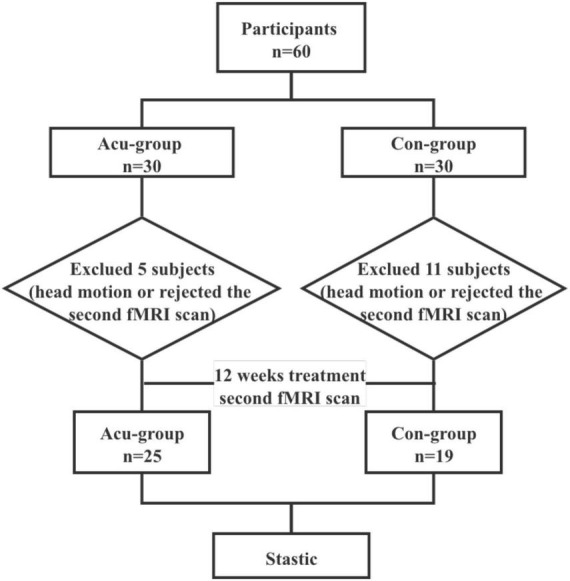
Workflow and group inclusion/exclusion criteria.

This study represents a randomized controlled trial using fMRI scans to assess the effect and mechanisms of acupuncture treatment on CSVDCI. Participants completed fMRI scans and cognitive function assessments at the baseline. We randomly divided the participants into two groups, one receiving acupuncture at the Shenting and Baihui acupoints combined with conventional treatment, and the other receiving conventional treatment only. After treatment, fMRI scans and cognitive function assessments were performed again (Figure 1). The acupuncture treatment lasted for approximately 40 min.

### 2.3. Blind

A random number generator with SPSS 22.0 statistical software was used by a researcher specializing in random assignment to derive 60 random numbers and generate a random assignment sequence. The cards with the random numbers, groupings, and interventions were then concealed in airtight, opaque envelopes and kept securely by this researcher. This person was not allowed to participate in the recruitment screening, outcome assessment and statistical analysis of this study.

### 2.4. Treatment program

#### 2.4.1. Acupuncture treatment

With the thumb and forefinger holding the needle handle, the doctor alternately twists the needle body clockwise and counterclockwise to make it rotate quickly (180–300 times/min), and continues twisting for 2–3 min. After this, twist once every 10 min (following Deqi), and keep the needle for 40 min. Participants received acupuncture treatment once a day, for five days a week over a total of 12 weeks of intervention. Our selected points are Shenting and Baihui. Shenting is on the head, 0.5 inch straight up from the middle of the front hairline. The Baihui point is located at the intersection of the median line at the top of the head and the line connecting the tips of the two ears. The acupuncture treatments were performed by Yang Xiaoyan, an associate chief physician who practices acupuncture for more than 10 years.

#### 2.4.2. Conventional treatment

Conventional treatment included donepezil tablets to improve cognition, aspirin to anti-platelet aggregation, atorvastatin calcium tablets to regulate lipid levels, in addition to blood pressure and blood glucose control according to the patient’s underlying disease, and each patient participated in modern cognitive rehabilitation training.

### 2.5. Cognitive assessment

Before and after the treatment, all participants completed a cognitive assessment. (i) The MoCA includes eight cognitive domains: visual-spatial and executive functions, naming, memory, attention, language, abstraction, delayed recall, and orientation. The total score and the score of each cognitive domain were recorded following previous studies (O’Driscoll and Shaikh, 2017). For education levels lower than 12 years, we added 1 point to the total score. (ii) SDMT (Silva et al., 2018): participants were asked to convert nonsensical symbols into numbers within 90 s, while we recorded the number of correct answers, which were given one point each. (iii) VFT (Sutin et al., 2019) consisted of three parts, including semantic, phonetic, and motor fluency. Participants were asked to say the corresponding words within one minute as required, and the sum of the three groups of correct numbers was the total score. (iv) DST (Leung et al., 2011) consisted of two parts, digit forward and digit backward. During the test, participants were asked to simultaneously remember two numbers read by the researcher, with one digit per second starting with the first set. (v) The BNT (Durant et al., 2021) test provided 30 graphs, with the number of correctly named graphs representing the total score. We also collected information on sociodemographic background, medication, and disease history. The cognitive assessors were Huang Xiaohuang and Ling Shuiqiao, both physicians are at the level of attending physician or higher and have at least 5 years of training in cognitive aspects of therapy.

### 2.6. Imaging data acquisition

All images were obtained using a GE 3T MRI scanner with an 8-channel phased-array head coil. The participants were requested to keep their eyes closed, relax but not fall asleep, and minimize head movement during the scanning. Functional images were collected with a gradient echo-planar imaging (EPI) sequence with the following parameters: repetition time (TR) = 2,000 ms, echo time (TE) = 30 ms, flip angle (FA) = 90°, field of view (FOV) = 240 mm × 240 mm, slice thickness = 3.5 mm, inter-slice gap = 0.7 mm, data matrix = 64 × 64, 33 interleaved axial slices coving the whole brain, and 240 volumes acquired in about 8 min. In addition, high resolution brain structural images were acquired using a T1-weighed 3D BRAVO sequence with the following parameters: TR = 8.0 ms, TE = 3.0 ms, FA = 12°, data matrix = 256 × 256, FOV = 256 mm × 256 mm, slice thickness = 1 mm, and 188 sagittal slices covering the whole brain. The conventional T1-weighted and T2-weighted FLAIR images were acquired for clinical assessment. All MRI images for each participant were acquired in the same session.

### 2.7. Data pre-processing

The fMRI data were preprocessed using the DPARSF toolbox^1^ based on MATLAB. Before pre-processing the data, we visually inspected both brain functional and structural images, and excluded the datasets with significant signal dropouts, distortion, and other quality problems. The pre-processing procedure included: (1) removing the first 10 volumes to keep the magnetization equilibrium; (2) performing slice-timing and head-movement correction to remove effects caused by these factors; (3) conducting a linear co-registration between functional and structural images for each participant; (4) regressing the signals of the WM and CSF, and head-movement parameters (Friston-24 model); (5) performing a non-linear transformation between structural and template brain images of the Montreal Neurological Institute (MNI) space; normalizing functional images into the MNI space with a 3 mm^3^ × 3 mm^3^ × 3 mm^3^ voxel size; and smoothing with a Gaussian kernel of 5 mm full width at half maximum (FWHM), and (6) performing temporal band-pass filtering for the typical band (0.01–0.08 Hz). This study discarded the fMRI data for subjects with head motion displacement > 3 mm or rotation > 3° in any axis (*x, y*, and *z*-axis). Data pre-processing was performed by Chen Sina.

### 2.8. ALFF analysis

We first performed voxel-wise Fast Fourier Transform (FFT) for each participant to convert the filtered time series into the frequency domain to obtain the power spectrum. Since the power at a given frequency is proportional to the square of the magnitude of that frequency component, we calculated the square root of the power spectrum at each frequency and the average square root in the typical frequency band (0.01–0.08 Hz) at each voxel. This averaged square root was taken as ALFF (Zang et al., 2007), which was assumed to reflect the absolute intensity of spontaneous brain activity.

### 2.9. Statistical analysis

#### 2.9.1. Demographic and cognitive assessment

A χ^2^-test was used to test between-group differences in gender. A *t*-test was used to test between-group differences in age. The Mann–Whitney U test was used to evaluate the education level, smoking, and alcohol consumption history between groups. The statistical significance level was set at *p* < 0.05. Statistical analysis was conducted using SPSS (version 22.0). Continuous variables of MoCA without normal distributions were analyzed using the Mann-Whitney U test. VFT, SDMT, BNT, and DST with normal distribution were analyzed using an independent *t*-test.

#### 2.9.2. ALFF and brain-cognitive correlation

The between-group differences test in ALFF was conducted using PALM and implemented in the DPARSF toolbox (see text footnote 1). In the calculations, a general linear model (GLM) was applied, and gender, education, and age factors were regressed. The significance level was set at *P* < 0.005. Permutation tests and multiple comparison corrections were applied to all statistics. Mixed effect analysis with a whole brain mask was utilized while examining group differences, which involved a comparison of the ALFF maps.

For each group, the ALFF maps were assessed using paired *t*-test. The significance level was set at a corrected two-tailed *P* value <0.05. Corrections for multiple testing were done using the threshold free cluster enhancement (TFCE) and family wise error (FWE) methods with the DPABI package.

Mean ALFF values of the obtained regions with significant group differences were extracted. Pearson’s correlation analysis was performed to examine the association between ALFF values and MoCA changes. All statistical analyses were performed using SPSS and a statistical significance level of *P* < 0.05.

## 3. Results

### 3.1. Demographics and acupuncture effects on cognition

Table 1 shows the demographic characteristics of all participants in each group. There were no significant differences in demographic variables between the two groups (*P* > 0.05). After statistical analysis, the results also showed no statistically significant differences in MoCA, SDMT, VFT, DST, and BNT scores between the two groups of subjects at the baseline level (*P* > 0.05). Further details are shown in Table 2. Compared with pre-intervention, the acupunture group showed better improved than the control group as measured by the MoCA, SDMT, VFT, DST, and BNT scores after intervention (Table 3).

**TABLE 1:** Demographics at the baseline.

Characteristics	Acu-group (*n* = 25)	Con-group (*n* = 19)	*P*-value
Gender (M/F)^a^	13/12	9/10	0.761
Age (in years)^b^	61.9 ± 4.56	62.94 ± 2.83	0.375
Education (in years)^b^	9.48 ± 2.94	8.68 ± 2.05	0.321
Smoking (often/sometimes/never)^c^	4/3/18	3/3/13	0.836
Alcohol (often/sometimes/never)^c^	4/3/18	3/5/11	0.429

Values are presented as the mean ± SD. M, male; F, female; SD, standard deviation. ^a^*p*-values for the Pearson chi-square test. ^b^*p*-values for two-sample independent t-test. ^c^*p*-values for the Mann–Whitney U test.

**TABLE 2:** Cognitive assessments at the baseline.

Characteristics	Acu-group (*n* = 25)	Con-group (*n* = 19)	*P*-value
MoCA^a^	22.00 (6.00)	22.00 (6.00)	0.319
SDMT^a^	52.00 (14.00)	51.00 (10.00)	0.859
VFT^a^	21.00 (8.00)	20.00 (5.00)	0.243
DST^b^	7.36 ± 1.97	6.47 ± 1.39	0.103
BNT^b^	17.96 ± 2.49	17.36 ± 2.38	0.431

MoCA, SDMT, and VFT values are presented as M(IQR); DST and BNT values are presented as the mean ± SD. M, median; IQR, interquartile range; SD, standard deviation. ^a^*p*-values for the Mann–Whitney U test. ^b^*p*-values for two-sample independent t-test.

**TABLE 3:** Group differences before and after treatment.

Characteristics	Acu-group (*n* = 25)		Con-group (*n* = 19)		*P*-value
	Rank	Percentage	Rank	Percentage	
MoCA	2.0 (2.0)	10.53%	1.0 (2.0)	6.55%	**0.015**
SDMT	4.0 (2.0)	8.15	2.0 (1.0)	2.23	**0.000**
VFT	3.0 (1.5)	15.56	2.0 (2.0)	9.72	**0.009**
DST	2.0 (1.0)	35.52	1.0 (2.0)	32.85	**0.005**
BNT	2.0 (2.0)	10.86	1.0 (2.0)	7.90	**0.015**

Values are presented as M(IQR); *P*-values for the Mann–Whitney U test. Percentage means “Pre- and post-treatment difference/Pre-treatment value”. M, median; IQR, interquartile range.

### 3.2. Acupuncture effects on regional functional activity

#### 3.2.1. Between-group analysis

**FIGURE 2:**
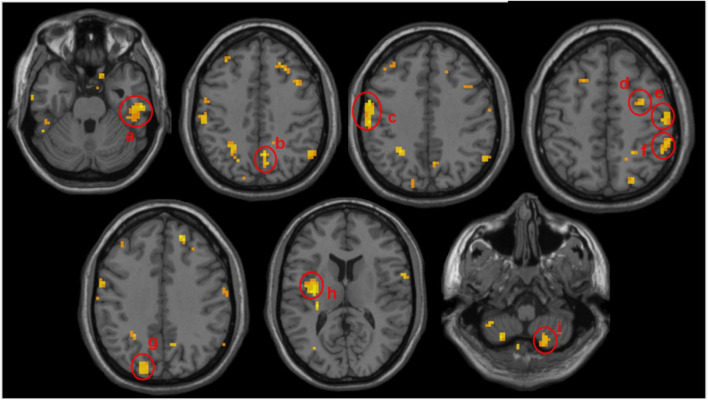
Volumetric results of the subtracted ALFF values mix effect analysis between the acupuncture and control groups. Subtracted ALFF values before and after the intervention was extracted separately from ACU and CON groups and mix effect analysis was performed to compute the difference for the treatment effect. Warmer colors represent higher ALFF changes in the acu-group compared to the con-group. Peak coordinates refer to the Montreal Neurological Institute (MNI) atlas. a, the right inferior temporal gyrus; b, the right precuneus; c, the left postcentral gyrus; d, the left precentral gyrus; e, the right postcentral gyrus; f, the right superior parietal gyrus; g, the left middle occipital gyrus; h, the left insula; i, the right cerebellum.

Comparing the ALFF differences before and after the intervention in the two groups, we found several brain regions with significantly higher ALFF values in the treatment group compared to controls, including the right inferior temporal gyrus, the left middle occipital gyrus, the left superior occipital gyrus, the left insula, the bilateral postcentral gyrus, the right superior parietal gyrus, the right cerebellum, the right precuneus, and the right precentral gyrus (*P* < 0.005). Details are shown in Figure 2 and Table 4.

**TABLE 4:** Comparison of the difference between the before and after ALFF values of the two groups.

Conditions	Cluster size	Brain region	Peak *t*-values	MNI		
				*X*	*Y*	*Z*
Con>Acu	None cluster					
Acu>Con	86	Temporal_Inf_R	4.00158	57	–21	–27
	81	Occipital_Mid/Sup_L	4.14463	–33	–72	15
	65	Insula_L	4.08554	–33	–9	12
	48	Postcentral_L	3.94765	–57	0	39
	45	Parietal_Sup_R	3.78277	27	–51	54
	39	Cerebellum_R	3.35485	18	–63	–57
	34	Precuneus_R	3.83540	6	–60	42
	31	Precentral_R	3.88859	54	3	27
	31	Postcentral_R	3.53786	66	–12	27

The significance threshold was set at *P* < 0.005 with no correction. Con-group (*n* = 19), acu-group (*n* = 25). Coordinates of the peak voxel are shown in the Montreal Neurological Institute (MNI) space. The *t*-value corresponds to the peak voxel with a significant between-group difference in ALFF. Inf, inferior; Mid, middle; Sup, superior; L(R), left (right) hemisphere.

#### 3.2.2. Longitudinal analysis

**FIGURE 3:**
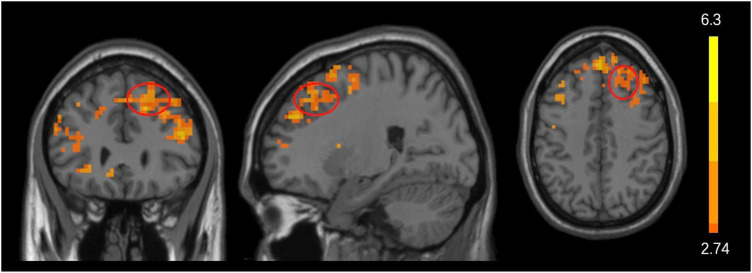
Acupuncture effects before and after treatment. Significant changes in ALFF values before and after acupuncture treatment in the acu-group. Significantly increased (marked in warmer colors) ALFF values were found in the frontal cortex after acupuncture treatment. The red circle represents the frontal lobe.

Paired *t*-test results (TFCE and FWE multiple comparisons corrected *P* < 0.05 and cluster size > 200 voxels) showed that, when compared with pre-treatment, acupuncture at Shenting and Baihui showed increased ALFF values in the bilateral middle/superior/inferior frontal gyrus and the left caudate and putamen (Table 5 and Figure 3). In the control group, we found no significant differences before or after treatment.

**TABLE 5:** Comparison of ALFF values before and after treatment in acu-group.

Group	Conditions	Cluster size	Brain region	Peak *t*-values	MNI		
					*X*	*Y*	*Z*
Acu-group	Post>Pre	2010	Frontal_Mid_R/L Frontal_Sup_R/L Frontal_Inf_R/L	6.3066	0	48	45
		231	Caudate_L/ Putamen_L	4.3959	–21	3	9

This result was achieved by comparing the ALFF maps before and after treatment. L, left; R, right; Mid, middle; Sup, superior; Inf, inferior.

### 3.3. Association between the changes in ALFF and MoCA after acupuncture

**FIGURE 4:**
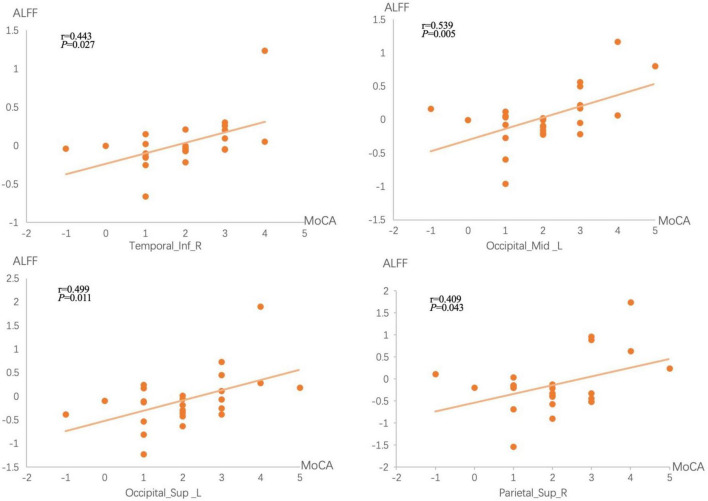
Correlations between brain measures and treatment performances. The *x*-axis represents the difference in MoCA scores before and after treatment, while the *y*-axis represents ALFF values differences before and after treatment.

Correlation analysis showed differences in ALFF values in the right inferior temporal gyrus, left middle occipital gyrus, left superior occipital gyrus, and right superior parietal gyrus in the acupuncture group were significantly positively correlated with change of MoCA (*P* < 0.05; Figure 4).

## 4. Discussion

We investigated cognitive function alterations (including MoCA, DST, VFT, SDMT, and BNT) in CSVDCI patients before and after acupuncture treatment, and performed neurology mechanisms voxel-based analysis of MRI-derived ALFF maps. According to our findings, and in contrast to conventional treatments, acupuncture at Shenting and Baihui significantly improved the cognitive function of patients. Importantly, we found an increase in spontaneous activity in regional brain areas, such as the right inferior temporal gyrus, left middle occipital gyrus, left superior occipital gyrus, and right superior parietal gyrus.

By comparing ALFF changes with those observed in the control group, we found that acupuncture combined with conventional treatment increased ALFF values of the right inferior temporal gyrus, left middle occipital gyrus, left superior occipital gyrus, left insula, bilateral postcentral gyrus, right superior parietal gyrus, right cerebellum, right precuneus, and right precentral gyrus in CSVDCI patients. This suggests that acupuncture at Shenting and Baihui may improve cognitive function by enhancing neuronal excitability in some brain regions of CSVDCI patients.

The inferior temporal gyrus is located in the temporal lobe. The structures in the medial temporal lobe, including the hippocampus, the internal olfactory and perirhinal cortex, and parietal hippocampal cortex, are important elements of long-term memory processing (Lech and Suchan, 2013). Wu et al. (2017) found that the combination of acupuncture and conventional treatment significantly improves motor and cognitive functions in stroke patients, and increased Reho values in the middle temporal gyrus. According to the authors, acupuncture may have a specific mechanism of action. PET technology showed that acupuncture points, such as Baihui, significantly increased glucose metabolism in the temporal and frontal lobes, improving cognitive function (Huang et al., 2007). Combined with our findings, the evidence supports that acupuncture of Shenting and Baihui significantly improve temporal lobe glucose metabolism levels, enhance energy supply, and increase local neuronal activity in CSVDCI patients, thus improving temporal lobe related cognitive functions.

Both the middle and superior occipital gyrus are part of the occipital lobe, an essential component of the visual center that transmits spatial information to the parietal lobe, which conveys the integrated spatial information to the prefrontal lobe, eventually forming spatial memory in the prefrontal area (Andersson et al., 2019). Previous studies found that the size of the white matter lesion volume in the occipital lobe in MCI patients is negatively correlated with cerebral blood flow, suggesting that decreased cerebral blood flow in the occipital lobe may lead to lesions in occipital lobe structures and to a decrease in cognitive function (Kim et al., 2020). According brain neuroimaging studies meta-analysis (Cao et al., 2020), the occipital lobe plays a role in the pathophysiology of dementia, suggesting it should be a target region for scalp acupuncture for treating dementia. Acupuncture of Shenting and Baihui improved executive function and visuospatial localization in CSVDCI patients, and their improvement was also correlated with improved spontaneous activity in the occipital lobe region.

The parietal cortex is an interesting part of the association cortex. Throughout modern neuroscience research, this region has been associated with a wide range of sensory, motor, and cognitive functions (Freedman and Ibos, 2018). Functional magnetic resonance imaging has been widely used to study the effects of acupuncture on neural activity. A study on functional MRI in MCI patients suggested that acupuncture increases functional connectivity between the parietal lobe and other cognitively relevant areas (Tan et al., 2017). Conversely, acupuncture increased Reho values of the parietal lobe in MCI patients. Hence, it is possible that that acupuncture also improves the regional homogeneity of different delicate structures in the parietal gyrus and increases spontaneous brain activity (Liu et al., 2014). Zhang J. et al. (2021) found that acupuncture reorganizes cognition-related brain areas, including the inferior frontal gyrus, and the temporal, parietal, and occipital lobes, and modulates post-stroke function and structural plasticity.

Acupuncture is widely used to cure post-stroke hemiplegia, cognitive dysfunction, anxiety, depression, among others (Wang et al., 2018; Du et al., 2020; Zhang et al., 2021). Li A. et al. (2018) explored the activating effects of acupuncture on the brain of healthy individuals using fMRI techniques and found it activates the postcentral gyrus, the precuneus, and the temporal and occipital lobes. Our results further validate these findings and reinforce the fact that acupuncture positively impacts spontaneous activity in various brain regions of CSDVDCI patients. Specifically, significant brain responses were observed after acupuncture stimulation at Shenting and Baihui, as well as improved ALFF values of the right inferior temporal gyrus, left middle occipital gyrus, superior occipital gyrus, and right superior parietal gyrus, which were positively correlated with an improvement in cognitive function.

## 5. Limitations

(1) The sample size was limited because this was a single-center study and screening for contraindications to MRI scanning was inadequate, resulting in some patients being unable to participate in the examination due to e.g., the presence of dentures, excessive head movement, and other factors. (2) In addition to cognitive dysfunction, CSVDCI patients also present with limb dysfunction, such as movement delays and mild hemiparesis, but our study did not evaluate such patients.

## 6. Conclusion

Acupuncture of Shenting and Baihui effectively improves cognitive brain function in CSVDCI patients. This may be related to an increase in spontaneous activity in local brain regions and changes in ALFF values at the right inferior temporal gyrus, left middle occipital gyrus, left inferior occipital gyrus, and left superior parietal gyrus.

## Data availability statement

The raw data supporting the conclusions of this article will be made available by the authors, without undue reservation.

## Ethics statement

The studies involving human participants were reviewed and approved by the Institutional Review Board of Zhongshan TCM hospital (ClinicalTrials.gov identifier: 2017ZSZY-LLK-219). The patients/participants provided their written informed consent to participate in this study. Written informed consent was obtained from the individual(s) for the publication of any potentially identifiable images or data included in this article.

## Author contributions

NY and LC designed the study. NY, SC, LC, SXL, and SQL collected the data. NY and SC analyzed the data and prepared the manuscript. All authors contributed to the article and approved the submitted version.

## Footnotes

1
http://rfmri.org/dpabi
